# Relationship between altitude and the prevalence of hypertension in Tibet: a systematic review

**DOI:** 10.1136/heartjnl-2014-307158

**Published:** 2015-05-07

**Authors:** Cuomu Mingji, Igho J Onakpoya, Rafael Perera, Alison M Ward, Carl J Heneghan

**Affiliations:** 1 Tibetan Medical College, Lhasa, Tibet & Austrian Academy of Sciences, Vienna, Austria; 2 Nuffield Department of Primary Care Health Sciences, Centre for Evidence-Based Medicine, University of Oxford, Oxford, UK

## Abstract

**Introduction:**

Hypertension is a leading cause of cardiovascular disease, which is the cause of one-third of global deaths and is a primary and rising contributor to the global disease burden. The objective of this systematic review was to determine the prevalence and awareness of hypertension among the inhabitants of Tibet and its association with altitude, using the data from published observational studies.

**Methods:**

We conducted electronic searches in Medline, Embase, ISI Web of Science and Global Health. No gender or language restrictions were imposed. We assessed the methodological characteristics of included studies using the Strengthening the Reporting of Observational Studies in Epidemiology (STROBE) criteria. Two reviewers independently determined the eligibility of studies, assessed the methodology of included studies and extracted the data. We used meta-regression to estimate the degree of change in hypertension prevalence with increasing altitude.

**Results:**

We identified 22 eligible articles of which eight cross-sectional studies with a total of 16 913 participants were included. The prevalence of hypertension ranged between 23% and 56%. A scatter plot of altitude against overall prevalence revealed a statistically significant correlation (r=0.68; p=0.04). Meta-regression analysis revealed a 2% increase in the prevalence of hypertension with every 100 m increase in altitude (p=0.06). The locations and socioeconomic status of subjects affected the awareness and subsequent treatment and control of hypertension.

**Conclusions:**

The results from cross-sectional studies suggest that there is a significant correlation between altitude and the prevalence of hypertension among inhabitants of Tibet. The socioeconomic status of the inhabitants can influence awareness and management of hypertension. Very little research into hypertension has been conducted in other prefectures of Tibet where the altitude is much higher. Further research examining the impact of altitude on blood pressure is warranted.

## Introduction

Hypertension is a leading cause of cardiovascular disease, which is the cause of one-third of global deaths and is a primary and rising contributor to the global disease burden.^1^

Exposure to hypoxia at high altitude is increasingly being recognised as a risk factor for hypertension.^2^ ^3^ However, studies investigating the relationship between high altitude and hypertension are scarce.^4^ Because altitude-related hypertension is primarily related to hypoxia, gaining an insight into the hypoxia–hypertension mechanism may help with management of hypoxia-related conditions. This study focused on Central Tibet where there is concentration of inhabitants living in high plateau.

Tibet is known as the ‘Third Pole’ and is one of the highest inhabited areas of the world. The average altitude is 4500 m above sea level. Life expectancy is stated at 67 years old,^5^ which is lower than Inland China by 8 years.^6^ However, the leading causes of death in Tibet have not been identified due to an absence of data. In addition, there are no reliable statistical results to illustrate healthcare service coverage, and the general infrastructure such as the condition of roads is still very poor, with severely sick patients often dying on their way to the nearest clinic or hospital due to serious limitations in overall living conditions.

Data from observational and genetic studies have shown that Tibet has a higher hypertension rate than in other Chinese areas,^7^ ^8^ as well as non-Chinese regions where inhabitants live at high altitudes.^9^ This leads to further serious comorbidities such as stroke, with a much higher prevalence than other Chinese areas.^10^ However, the health surveys on the prevalence of hypertension in Tibet are limited, as the currently available evidence only indicates data collected mainly from the City of Lhasa (the capital of Tibet) and nearby counties, while hardly any research has been conducted during the last three decades in other regions of Tibet, where there are higher altitudes and more harsh living conditions.

Until now, causative studies have mainly focused on two factors: the special environmental features of Tibet—the high altitude, and the relatively unique lifestyle led by indigenous people, looking specifically at diet. With the latter, significantly higher sodium levels were found among Tibetan subjects compared with other ethnic groups in China.^11^ Contradictory theories have been proposed regarding the mechanistic pathway of blood pressure (BP) regulation on exposure to high altitudes. Some authors have hypothesised that exposure to high altitude results in increased BP^12^ ^13^ possibly due to greater autonomic or sympathetic activities,^14^ while other investigators have postulated that the initial elevation observed on exposure to high altitude normalises and even drops below what is observed at sea level, after years of habitation at high altitudes.^15–19^ Evidence from previous research has also suggested an increased prevalence of hypertension at high altitudes,^20^ ^21^ possibly due to greater stimulation of the chemoreceptor reflex in individuals with borderline hypertension.^14^ ^16^

Despite variable hypotheses around the correlation between high altitude and hypertension, there has not been a systematic review of the studies of hypertension in Tibet. This has resulted in the lack of a commonly identifiable conclusion on the correlation between high altitude and hypertension. Therefore, the objective of this systematic review is to determine the prevalence and awareness of hypertension among the inhabitants of Tibet and its association with altitude, using the data from published observational studies.

## Methods

We conducted electronic searches of the following databases: Medline, Embase, ISI Web of Science and Global Health. Each database was searched from inception until February 2014. Search terms used included blood pressure, hypertension, prevalence, altitude, Tibet and derivatives of these (a comprehensive Medline search strategy used has been included as an appendix) (see online supplementary appendix 1). We also searched the bibliographies of all located articles and no gender or language restrictions were imposed.

We included cross-sectional and observational studies. For inclusion, studies had to report the prevalence of hypertension among residents ≥15 years old domiciled in any Tibetan province as an outcome. Studies not conducted in Central Tibet or Tibetan provinces of China were excluded from the review. We assessed the methodological characteristics of included studies using a checklist adapted from the Strengthening the Reporting of Observational Studies in Epidemiology (STROBE) criteria.^22^ Two reviewers (CM and IJO) independently determined the eligibility of studies, assessed the methodology of included studies and extracted the data. Disagreements were resolved through consensus.

We extracted data according to study design, setting and altitude, participants’ demographics, socioeconomic status, diagnostic criteria and the prevalence of hypertension, awareness, treatment and control rates of hypertension. Summary tables were used to report the methodological quality and characteristics of main results of included studies, while scatter plots were used to explore the relationship between altitude and the prevalence of hypertension. Meta-regression statistics using the Stata statistical package (V.13) was used to investigate the degree of change in the prevalence of hypertension with increasing altitude. We also performed meta-regression based on imputed mean age using either the one given or by imputation from studies with similar age range.

## Results

**Figure 1:**
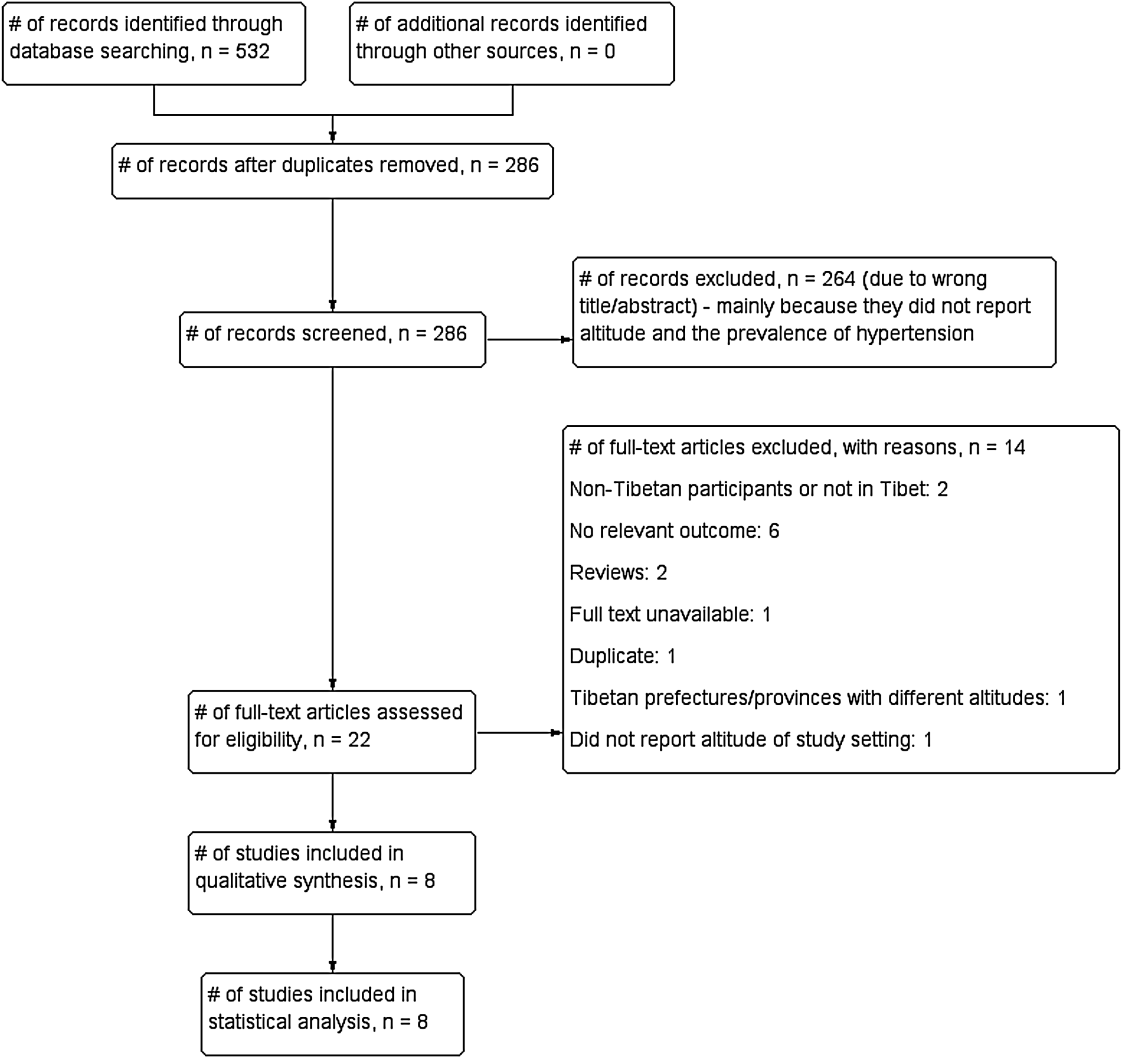
Flow diagram showing process for the inclusion of observational studies exploring the association between altitude and the prevalence of hypertension among Tibetan inhabitants.

The initial search of data identified 286 potential articles (figure 1). After scanning the titles and abstracts, 22 articles were eligible for full textual review. Among these, six were excluded due to having no relevant outcome;^16^ ^23–26^ two because they presented research which was either not conducted in Tibet^27^ or where the targeted subjects were non-Tibetans;^28^ two due to being review papers;^10^ ^29^ one because the full text could not be retrieved;^30^ one^31^ due to being a duplicate of another study included in the review; one^7^ because it was conducted across settings with different altitudes and one because it did not report the altitude of the study setting.^32^ This left eight studies with a total of 16 913 participants for inclusion in the review.^11^ ^33–39^

There were some discrepancies in the reporting characteristics of the included studies (table 1). All eight were cross-sectional in design. Three used stratified sampling methods to recruit participants,^33–35^ three used simple randomised sampling^11^ ^36^ ^38^ and one study used mixed methods for sampling.^39^ The sampling method used in one study was not specified.^37^ The sample size for respondents in all studies was representative of the defined populations; across studies, the response rates ranged between 65% and 100%.

**Table 1:** Quality assessment of the included studies examining the relationship between altitude and the prevalence of hypertension

Study ID	Study design	Definition of hypertension	Definition of awareness	Definition of treatment	Definition of control	Sampling strategy	Response rate	Adequate sample size	Validated measure	Appropriate statistical analysis
Sherpa^33^	Cross-sectional	≥130/85 mm Hg or antihypertensive therapy	Self-report of prior diagnosis by a healthcare professional	Self-reported use of a prescribed medication	Medication treatment with BP <140/90 mm Hg	Stratified	82%	Yes	Yes	Yes
Zhao^34^	Cross-sectional	≥140/90 mm Hg or antihypertensive therapy	Self-report of prior diagnosis by a healthcare professional	Self-reported use of a prescribed medication	Medication treatment with BP <140/90 mm Hg	Stratified	100%	Yes	Yes	Yes
Zheng^35^	Cross-sectional	≥140/90 mm Hg or antihypertensive therapy	Self-report of prior diagnosis by a healthcare professional	Self-reported use of a prescribed medication during last 2 weeks	Medication treatment with BP <140/90 mm Hg	Stratified	96.8%	Yes	Yes	Yes
Deji^36^	Cross-sectional	≥140/90 mm Hg or antihypertensive therapy	Self-report prior to the survey	Self-reported use of a prescribed medication during last 2 weeks	Medication treatment with BP <140/90 mm Hg	Randomised	65%	Yes	Yes	Yes
Lei^37^	Cross-sectional	≥140/90 mm Hg	Self-report prior to the survey	Self-reported use of a prescribed medication during last 2 weeks	Medication treatment with BP <140/90 mm Hg	Not reported	100%	Yes	Yes	Yes
Zhang^38^	Cross-sectional	≥140/90 mm Hg or proof of hospital diagnosis	Not reported	Not reported	Not reported	Randomised	89%	Yes	Yes	Yes
Liu^11^	Cross-sectional	≥140/90 mm Hg or antihypertensive therapy	Not reported	Not reported	Not reported	Randomised	100%	Yes	Yes	Yes
Li^39^	Cross-sectional	≥140/90 mm Hg	Not reported	Not reported	Not reported	Random, cluster and multistage	87%	Yes	Yes	Yes

The definition of hypertension was consistent across seven studies (≥140/90 mm Hg); one study^33^ used ≥130/85 mm Hg as cut-off criteria (table 1). Awareness was defined as self-report of prior diagnosis of hypertension by a healthcare professional (three studies) and self-report prior to the survey (two studies). Three of the included studies did not define the criteria for awareness. Five studies defined treatment of hypertension as self-reported use of medication prescribed by a healthcare professional, while three studies did not define hypertension treatment. Five of the included studies defined control of hypertension as medication use resulting in BP <140/90 mm Hg.

The settings varied between studies. Four studies were conducted using a mixture of rural and urban areas,^34^ ^35^ ^38^ ^39^ three in urban^11^ ^36^ ^37^ and one exclusively in rural areas.^33^ The age of participants across the studies was between 15 and 90 years. Only three studies reported socioeconomic status; one of these was conducted in an urban area and included professionals, whereas the other studies included either herdsmen and/or farmers as participants (table 2).

**Table 2:** The main results of studies examining the altitude, prevalence and awareness of hypertension among Tibetan inhabitants

Study ID	Region	Study setting	Age (years)	Sample size	Altitude (m)	Prevalence of hypertension (%)			Social status	Awareness rate	Treatment rate	Control rate	Prevalence–age relationship
						Overall	Men	Women					
Sherpa^33^*	Tibetan Autonomous	Rural	30–80	766	3700	32.5	32.8	32.2	Farmers and herdsmen	69.4%	59.1%	19.5%	Not reported
Zhao^34^	Tibetan Autonomous	Rural/urban	≥40	702	4300	55.9	66.1	48.3	Herdsmen	19.9%	2.6%	0.3%	Sig ↑in prevalence with ↑d age, p=0.01
Zheng^35^*	Tibetan Autonomous	Rural/urban	≥18	1416	3650	51.2	56	48	Not reported	63.5%	24.3%	31.8%	Sig ↑in prevalence with ↑d age, p=0.001
Deji^36^*†	Tibetan Autonomous	Urban	30–70	571	3650	40.2	36.6	40.9	Urban residents and professionals	70%	38.1%	2.4%	Sig ↑in prevalence with ↑d age, p=0.001
Lei^37^†	Tibetan area, Sichuan	Urban	21–72	284	4000	32.7	Not reported	Not reported	Not reported	26.9%	9.7%	4.3%	Sig ↑in prevalence with ↑d age, p=0.05
Zhang^38^†	Tibetan area, Sichuan	Rural/urban	18–90	5049	3500	22.9	25.4	20.6	Not reported	Not reported	Not reported	Not reported	Sig ↑in prevalence with ↑d age, p=0.01
Liu^11^	Tibetan Autonomous	Urban	48–56	125	3760	39.75	29	51	Not reported	Not reported	Not reported	Not reported	Not reported
Li^39^*†	Tibetan area, Gansu	Rural/urban	≥18	8000	3000	24.6	25	23	Not reported	30.4%	20.7%	5.5%	Not reported

*Study conducted in Lhasa with different altitudes; data excluded from statistical analysis.

†Article written in Chinese.

**Figure 2:**
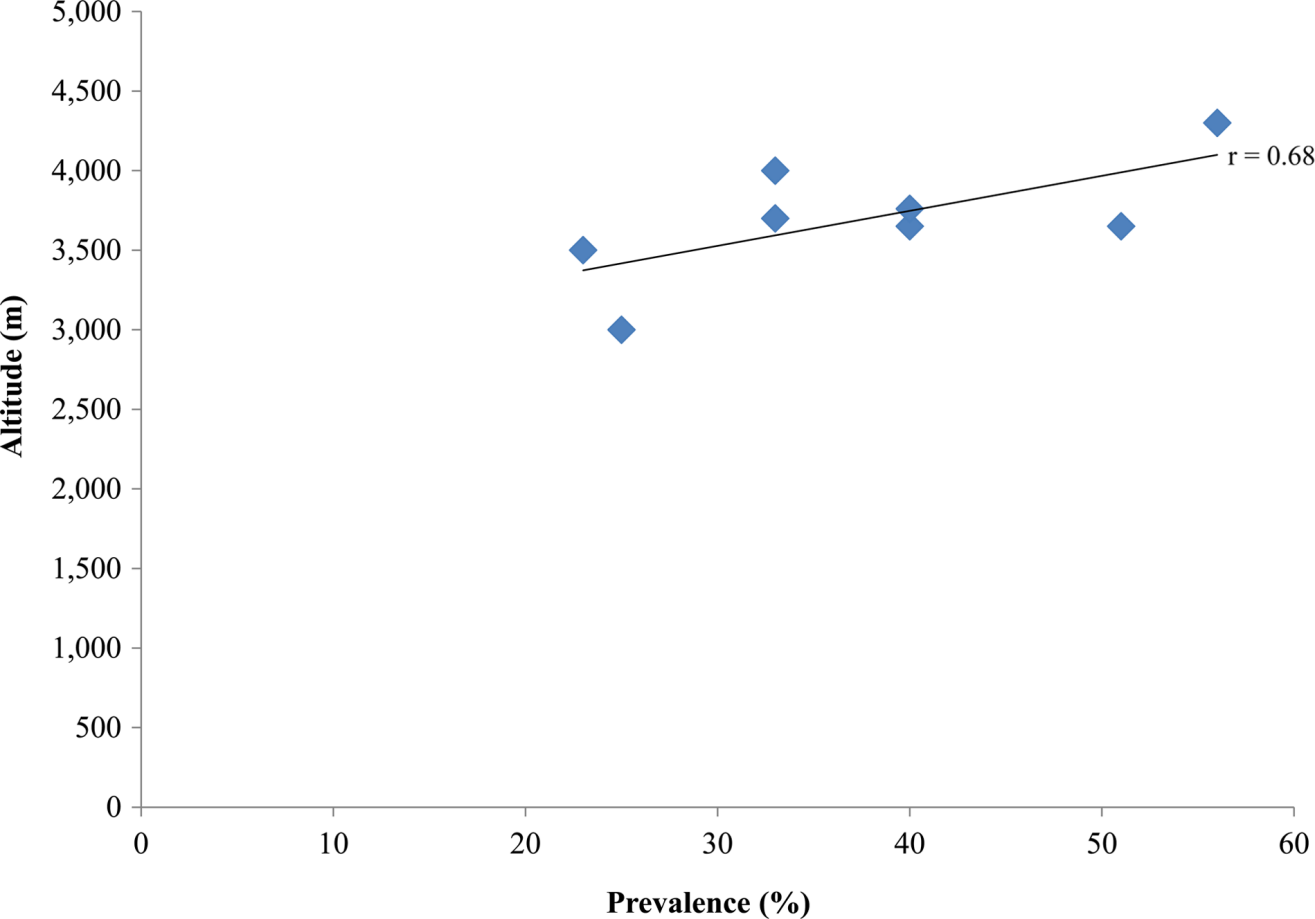
Relationship between altitude and the prevalence of hypertension. The p value of the relationship was 0.04.

**Figure 3:**
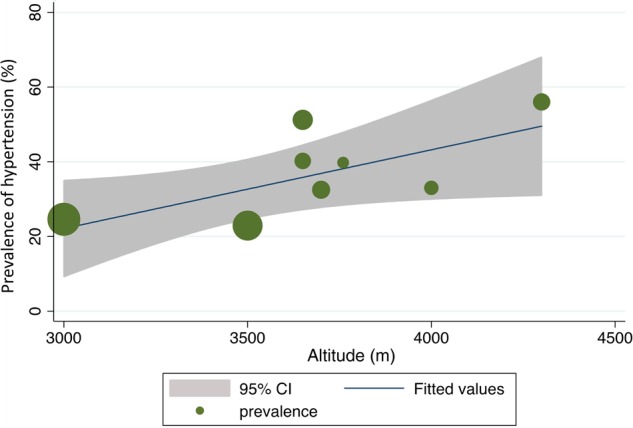
Meta-regression analysis showing the relationship between altitude and prevalence. The sizes of the circles correspond to the sample sizes of the included studies. There was a near significant relationship between altitude and prevalence (p=0.06). An increase of 1 m in altitude was associated with a 0.02% increase in the prevalence of hypertension.

**Figure 4:**
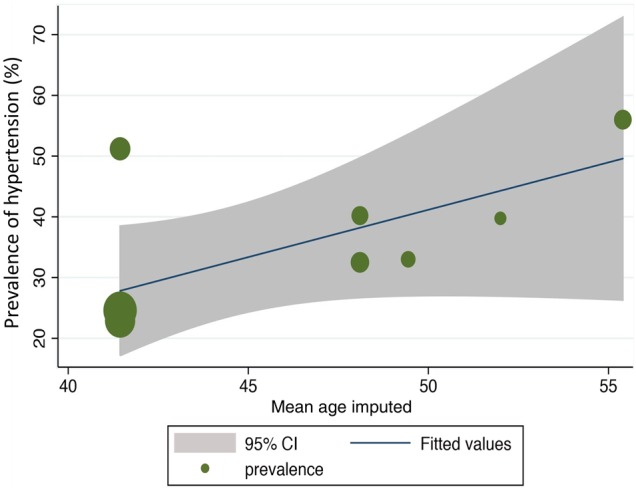
Meta-regression analysis showing the association between altitude, mean age and the prevalence of hypertension. There was a trend towards increased prevalence of hypertension with increasing altitude irrespective of age (p=0.06). The meta-regression was based on imputed mean age using either the one given or imputing from a study that had similar age range (in one case using the median age for the age range reported). It was not possible to use age as a confounder due to inadequate data. The sizes of the circles correspond to the sample sizes of the included studies.

The altitudes ranged from 3000 to 4300 m, and the prevalence of hypertension ranged between 23% and 56% (table 2). A scatter plot of altitude against overall prevalence excluding this study (figure 2) showed a statistically significant correlation (r=0.68; p=0.04). Meta-regression revealed that an increase in altitude of 1 m correlates with a 0.02% increase in prevalence (p=0.06; figure 3). Meta-regression based on imputed mean age using either the one given or imputing from a study that had similar age range did not reveal any observed change in the association (figure 4). However, there was insufficient data to explore the influence of age as a confounder.

Among the studies that used same standards of diagnostic criteria and had similar age range, the lowest hypertension prevalence was in Ganzi County, Sichuan Province, at 23%,^38^ whereas the highest prevalence was at 56% in Yangbajing–Dangxiong, Lhasa, which has the highest altitude at 4300 m.^34^

Four studies were conducted in the City of Lhasa or the surrounding countryside administered under the Lhasa Municipality.^11^ ^33^ ^35^ ^36^ Though a scatter plot suggested a trend towards increased prevalence of hypertension with increasing altitude in these studies, the relationship did not achieve statistical significance (r=0.65, p=0.17). Studies conducted in different Tibetan areas of Sichuan (Ganzi and Litang Counties)^37^ ^38^ which had no marked differences in dietary habits also showed similar trends (data not shown).

Five studies reported data on the association between age and hypertension. All reported a significant increase in prevalence with increasing age: two reported p values <0.001,^35^ ^36^ another two reported p<0.01;^34^ ^38^ while one reported p<0.05.^37^ One study^35^ reported significantly higher hypertension prevalence rates for urban, suburban and agricultural areas compared with pastoral areas (p<0.001); no other study compared prevalence rates between urban and rural settings.

Of the studies that reported hypertension prevalence according to gender, the prevalence of hypertension between male and female subjects differed over locations and age ranges, with prevalence ranging from 25.0% to 66.10% for men and from 20.6% to 51.0% for women. Three studies showed women as having greater hypertension prevalence, while the other five studies indicated men as having a higher prevalence (table 2). However, the differences in most studies were not apparent, while the prevalence result was noticeably higher in the female group at 51% versus male group at 29% in one particular study conducted in Lhasa.^11^ Separate scatter plots of altitude against prevalence for men and women did not reveal significant correlations between altitude and the prevalence of hypertension (p=0.13 and p=0.18, respectively), suggesting that gender may not have an influence on the prevalence of hypertension at high altitude.

Six studies reported on the rates of awareness, treatment and control of hypertension.^33–37^ ^39^ The highest rate of awareness was 70% in the City of Lhasa, where the socioeconomic status of subjects is at the level of urban residents/professionals,^35^ with the lowest rate being 20% in Yangbajing Town, Dangxiong County, Lhasa Municipality, where the socioeconomic level is that of herdsmen.^33^ The highest treatment rate (59%) was in rural Lhasa,^32^ with the lowest treatment rate reported at Yangbajing Town (2.6%).^33^ Lhasa also had the highest control rate (31.8%);^34^ this study included data collected from both the city and nearby rural areas. The lowest control rate (0.3%) was reported in Yangbajing Town.^33^ Apart from the studies conducted in Lhundub and Qushu Counties of Lhasa, all the other studies conducted in rural areas showed lower awareness, treatment and control rates.^33^ ^36^ ^39^

## Discussion

Our results indicate that there is a significant correlation between altitude and the prevalence of hypertension among inhabitants of Tibet, corresponding to a 2% increase in prevalence for every 100 m increase in altitude. The results of our review also show that the locations and socioeconomic status of subjects affect the awareness and subsequent treatment and control of hypertension. The results from five of the included studies corroborate the findings from previous research indicating an age-related increase in the prevalence of hypertension in Tibet.^40^ Our results are also consistent with the findings of a recent randomised clinical trial which reported progressive increases in both conventional and ambulatory BPs with increasing altitude.^41^ To our knowledge, this is the first systematic review which examines the relationship between altitude and the prevalence of hypertension among Tibetan inhabitants.

The evidence of the impact of altitude on BP is controversial.^42^ Researchers have previously attributed high altitude as a possible contributor to hypertension, and have suggested conducting of in-depth studies examining the correlation between altitude and BP.^43^ It has been suggested that chronic exposure to high altitude results in increased sympathetic and parasympathetic activities, which lead to raised BP.^44^ ^45^ However, there is very little research investigating the relationship between genetic adaptations and the prevalence of hypertension among Tibetan populations. According to Li and colleagues,^8^ two regulatory genes, the C allele at rs2070744 of the NOS3 and the T allele of rs4961 of the ADD1 gene, are significantly associated with the high prevalence of hypertension among Tibetan patients. Of these, the NOS3 gene was indicated as having a role in regulating vascular tone and blood vessel diameter, which may be altered by the low-oxygen environment of Tibet.

Though we found no differences in dietary lifestyle across the Tibetan regions, the influence of this variable on the prevalence of hypertension cannot be overlooked. Tibetans have been reported to consume ‘traditional salty butter tea’ whose salt content is four to five times the amount recommended by the WHO.^7^ ^46^ Indeed, the result of a recent study showed that a low-sodium, high-potassium salt-substitute intervention caused a dramatic fall in BP in hypertensive Tibetans.^47^

Even with the results of many studies illustrating that over time Tibetans can develop a certain degree of adaptation ability, such as greater forced vital capacity, lung diffusing capacity and blood haemoglobin concentration compared with lowland inhabitants,^48^ this does not necessarily imply that Tibetans do not experience hypoxia environmental challenge. The reduction of partial pressure gradients makes gas exchange more difficult, resulting in a long-term chronic insufficiency in the oxygenated blood circulation. This causes a drop in blood supply to the kidneys, and the subsequent release of renin that contributes to the vasoconstriction of the arteries.^49^

In terms of the influence of socioeconomic status on the awareness, treatment and control rates of hypertension, it is difficult to ascertain whether this is due to individuals’ better education or due to levels of accessibility to local healthcare services, and thus further studies are required due to an absence of data. The contradictory results demonstrated by the study conducted in Lhasa Municipality, which reported the highest awareness and treatment rates (70% and 38%), but with a very low control rate (2%) may indicate the stress impact of hypertension in urban dwellers; this has been reported by other authors.^50^ Apart from the studies conducted in Lhundub and Qushu Counties, Lhasa reported a highest treatment rate of 59%, and all other studies conducted in rural areas showed lower awareness, treatment and control rates. This may suggest that better health services are available in Lhundub and Qushu than in Yangbajing, due to distance and better transport facilities. The higher prevalence rates among herdsmen compared with multi-professionals in the Tibetan Autonomous regions (56% vs 40%) illustrate the ‘Inverse Care Law’ where the availability of medical care inversely varies with the population served.^51^

The differences in the prevalence of hypertension between genders were minor and no particular trend emerged. A higher prevalence was found among women compared with men in one study.^11^ However, in another study with similar participant characteristics (albeit with a larger sample size),^34^ the reverse was the case. Further research investigating whether gender is a risk factor for hypertension at high altitude will help remove this uncertainty.

### Strengths and limitations

This review has some strengths. It is the first which synthesises the information on the relationship between altitude and the prevalence of hypertension in Tibet. We employed a robust search strategy, and the included studies were generally of good reporting quality. The consistency observed in most of the relationships explored also suggests that altitude has a role in the pathogenesis of hypertension among Tibetans. However, we recognise some limitations. The included studies were all cross-sectional, and therefore not ideal for establishing causality. The review results are also only applicable to Tibetan populations. We also may not have identified all studies examining the relationship between altitude and the prevalence of hypertension among inhabitants of Tibet. In addition, the results could have been confounded by the variations in age, non-response bias and inconsistencies in awareness, treatment and control. Furthermore, the reporting of the data was not ideal; therefore, individual patient data would better have allowed us to explore the relationship between altitude and BP adjusted for other important confounders such as age and sex.

### Implications for research and practice

Most published surveys conducted so far are mainly concentrated in Lhasa, the capital city of Tibet, and surrounding counties, and rarely has research been carried out in other prefectures of Tibet where the altitude is much higher (≥4500 m) and living conditions harsher. Thus, more health surveys would provide an in-depth view of the relationship between high altitude and hypertension. In addition, further research is needed to identify the underlying mechanisms of why exposure to high altitude could lead to variable results. To achieve this, it may be important to consider the impact of altitude on hypertension in relation to its variable pathological states to identify an in-depth causality which is applicable to wider populations.

The significant correlation between altitude and the prevalence of hypertension in our review could help to identify high altitude as an important cause of hypertension at high altitude. This finding may provide new insights into the prevention and treatment of hypertension at high altitudes, resulting in a reduction of subsequent cardiovascular problems (eg, stroke and ischaemic heart disease), thereby increasing the overall life expectancy of high-altitude dwellers; indeed, research evidence suggests that commonly used BP medications may be ineffective at high altitudes.^41^ In addition, better understanding of the altitude–hypertension relationship may help provide novel techniques for managing conditions such as hypoxia of obstructive sleep apnoea which mimic some aspects of altitude.

## Conclusion

The results from cross-sectional studies suggest that there is a significant correlation between altitude and the prevalence of hypertension among inhabitants of Tibet. The results of our review also show that the locations and socioeconomic status of subjects can influence awareness and subsequent treatment and control of hypertension. Very little research into hypertension has been conducted in other prefectures of Tibet where the altitude is much higher. Further research examining the impact of altitude on BP among Tibetan inhabitants is warranted.

**Key messages**

**What is already known on this subject?**

- The results of genetic and observational research have shown that Tibet has a higher hypertension rate than in other Chinese areas.
- There is uncertainty about the relationship between high altitude and hypertension, and various hypotheses have been proposed.

**What might this study add?**

- There is a statistically significant correlation between altitude and the prevalence of hypertension in Tibet.
- An increase of 100 m in altitude is associated with a 2% increase in the prevalence of hypertension among Tibetan inhabitants.
- Socioeconomic status may influence the rates of awareness and subsequent control and treatment of hypertension.

**How might this impact on clinical practice?**

- Living at high altitudes could be a potential risk factor for hypertension.

## Supplementary Material

Web supplement
