# Supplementary material for: Relationship between altitude and the prevalence of hypertension in Tibet: a systematic review
Source: Heart. 2015 May 7;101(13):1054–60. doi: 10.1136/heartjnl-2014-307158 (PMC4484261; doi:10.1136/heartjnl-2014-307158)
Supplement: Web supplement [file heartjnl-2014-307158-s1.pdf]

Appendix 1: Search strategies

Embase: 10th February 2014

1	(tibet* or lhasa).ti,ab.	3503
2	(tibet* or lhasa).in.	909
3	1 or 2	3948
4	exp Hypertension/	490818
5	exp Antihypertensive Agent/	577323
6	Blood Pressure/	216946
7	(hypertensi* or antihypertensi* or anti-hypertensi*).ti,ab.	453993
8	blood pressure.ti,ab.	290530
9	4 or 5 or 6 or 7 or 8	1255031
10	3 and 9	179

Global Health: 10th February 2014

1	(tibet* or lhasa).ti,ab.	1127
2	(tibet* or lhasa).in.	123
3	1 or 2	1176
4	exp Hypertension/	21386
5	exp Antihypertensive Agents/	1457
6	Blood Pressure/	18312
7	(hypertensi* or antihypertensi* or anti-hypertensi*).ti,ab.	30224
8	blood pressure.ti,ab.	25215
9	10 or 11 or 12 or 13 or 14	47796
10	3 and 9	38

Global Health Library: 10th February 2014

(tibet OR tibetan OR tibetans OR tibeto OR lhasa OR qinghai OR sichuan OR yunnan OR gansu) AND (hypertension OR hypertensive OR "blood pressure" OR antihypertensive OR antihypertensives OR anti-hypertensive OR anti-hypertensives)

Medline: 10th February 2014

1	Tibet/	1064
2	(tibet* or lhasa).ti,ab.	2950
3	(tibet* or lhasa).in.	270
4	1 or 2 or 3	3183
5	exp Hypertension/	204972
6	exp Antihypertensive Agents/	222270
7	Blood Pressure/	231890
8	(hypertensi* or antihypertensi* or anti-hypertensi*).ti,ab.	319301
9	blood pressure.ti,ab.	214450

10	5 or 6 or 7 or 8 or 9	722320
11	4 and 10	112

Web of Science Core Collection: 10th February 2014
Indexes=SCI-EXPANDED, SSCI, A&HCI, CPCI-S, CPCI-SSH, BKCI-S, BKCI-SSH, CCR-EXPANDED, IC
Timespan=All years

# 3	138	#2 OR #1
# 2	45	**ADDRESS:** *(tibet* OR lhasa) AND* **TOPIC:** *(hypertensi* OR "blood pressure" OR antihypertens* OR anti-hypertens*)*
# 1	129	**TOPIC:** *(tibet* OR lhasa) AND* **TOPIC:** *(hypertensi* OR "blood pressure" OR antihypertens* OR anti-hypertens*)*
